# Optimization of the Dark Fermentation Technique for Hydrogen Production through Supplementation with Ascorbic Acid and/or l‑Cysteine by Clostridium butyricum CCDBC 11

**DOI:** 10.1021/acs.jafc.5c03194

**Published:** 2025-05-26

**Authors:** Hana Pistekova, Miroslava Dusankova, Tomas Sopik, Jakub Klaban, Jitka Dostalkova, Robert Moucka, Vladimir Sedlarik

**Affiliations:** Centre of Polymer Systems, University Institute, 561989 Tomas Bata University in Zlin, tr. Tomase Bati 5678,760 01 Zlin, Czech Republic

**Keywords:** biohydrogen production, dark fermentation, ascorbic acid, l-cysteine, *Clostridium butyricum*

## Abstract

This study explores the enhancement of biohydrogen production through the addition of oxygen scavengers, ascorbic acid, and l-cysteine during dark fermentation by Clostridium butyricum strain. The supplementation of these compounds significantly reduced the bacterial lag phase and accelerated cell growth, thereby boosting the hydrogen output. Using saccharified corn scrap as the substrate, a maximum cumulative hydrogen yield of 2.20 mol H_2_/mol glucose was achieved with 5 mg/L ascorbic acid. This treatment reduced the lag phase by 65.6% and increased the hydrogen yield by 40.9% compared to the control and by 11.4% relative to l-cysteine supplementation alone. Biogas production was quantified via the water displacement method, and hydrogen content was analyzed using gas chromatography. The results indicate that ascorbic acid is a cost-effective and efficient additive for improving the hydrogen yield in dark fermentation processes.

## Graphical abstract

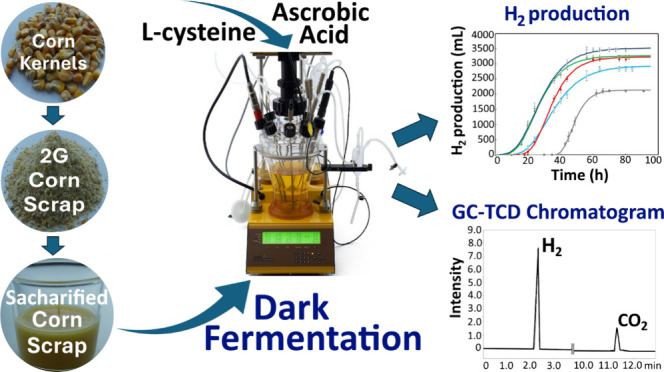

## 1 Introduction

Hydrogen is gaining global attention as a unique energy solution and a potential carbon-free fuel.^1^ Various conventional methods have been employed to enhance hydrogen production, with biological hydrogen production playing a significant role.^2^ This approach involves producing hydrogen through the cultivation of microorganisms,^3^ potentially via the biotechnological conversion of biomass.

Hydrogen production from organic waste biomass can generally be classified into two main processes: photosynthetic and dark fermentation. The latter offers distinct advantages, as it does not require light as an energy source, unlike photofermentation, and operates effectively with a simple reactor while achieving a higher hydrogen production rate. Moreover, a wide range of renewable biomass and organic waste materials are applicable as the substrate, thereby lowering the cost involved.^2–6^

The choice of substrate has a demonstrable effect on the fermentation process, in accordance with the biodegradability of the material. Glucose, maltose, and xylose are readily used, whereas others, e.g., starch, necessitate a preliminary transformation into glucose or maltose, either by acid or by enzymatic hydrolysis.^5^ Several carbohydrate-rich substrates are also suitable, including first-generation fuel crops, such as sugar cane, wheat, corn, and sugar beet, as well as second-generation (2G) biomass sources like agricultural residues, industrial waste, and wastewater.^7,8^ The use of 2G raw materials as a potential energy source for hydrogen production has gained significant interest due to their sustainability and potential to enhance the comprehensive utilization of renewable energy resources.^9^

Various microorganisms are capable of producing hydrogen, and notable among them are strict anaerobes, which cannot grow in the presence of oxygen. These microorganisms do not perform oxidative phosphorylation. Instead, the generation of adenosine triphosphate (ATP) primarily transpires via substrate phosphorylation and a flavin-based electron bifurcation process during fermentation. Strict anaerobes with the ability to produce hydrogen include Gram-positive bacteria of the genus *Clostridium.* ^3^ Although the maximum theoretical yield of hydrogen is 4 mol/mol glucose, the highest such values for the genus *Clostridium* reported in the literature are below 3 mol H_2_/mol glucose.^3,10^

The efficiency of dark fermentation is associated with the activity of hydrogenase and nicotinamide–adenine dinucleotide (NAD+/NADH), whose activity is supported by an environment with a low oxidation–reduction potential (ORP). Some reducing amino acids contribute to inhibiting such an ORP,^5,11^ notably l-cysteine, a low-cost reducing agent. The diminishing effect of l-cysteine on the oxidation–reduction potential of a fermentation system is exerted through the presence of a thiol group.^12^ This particular amino acid has also been described as a mediator between the given fermentative bacteria and substrate owing to its unique structure and affinity for certain bacterial proteins.^4^ Additionally, the disulfide bond (−S–S−), which can be formed from the thiol group of l-cysteine, plays a crucial role in protein formation. Furthermore, the remaining thiol groups may contribute to maintaining the protein structure, thereby regulating cellular metabolism.^13^ This enables it to function as a bioactive agent, enhancing the growth of fermenting bacteria and supporting substrate utilization.^14^

The influence of l-cysteine on hydrogen production has been investigated in several studies,^4,12,15–18^ wherein applying it shortened the lag phase and enhanced hydrogen production. In terms of the latter, Guo et al. reported an increase of 23.7% by adding 0.5 g/L l-cysteine to an expanded granular sludge bed.^19^ Zhang et al. recorded dark fermentative production as 1.6–2.0 times higher from cassava residues with the amino acid than from a control group without it (0.5–2.0 g/L).^16^ Yuan et al. described how supplementation with 0.6 mM l-cysteine increased the yield of hydrogen by 18.3%.^4^ Another study by Qu et al. reported a reduction in reactor residence time to 21 days, which was 4 days shorter than the blank sample, while daily hydrogen production increased by 3.2%.^17^ Additional examples are summarized by Yang and Wang in their review.^14^

Although most studies emphasize the beneficial effect of l-cysteine on hydrogen production, a manuscript by Zhao et al. reported a decrease in such from the Clostridium beijerinckii strain. Therein, a drop of 1.73–1.46 mol/mol sucrose (−15.6%) was observed upon the addition of 0.1 g/l-cysteine.^15^ For this reason, it seemed advisable to determine the outcome of applying l-cysteine to each strain.

In recent years, the use of ascorbic acid as an oxygen scavenger in the food industry has gained attention, both as a food additive and as a component of food packaging.^20,21^ Although its beneficial effect on promoting the growth of anaerobic microorganisms, particularly lactic acid bacteria, has been proven,^20^ few studies have investigated its effect on hydrogen production. An example by Zhu et al. detailed this aspect in photofermentation experiments;^22^ however, to our knowledge, no dark fermentation studies have been published.

Biological hydrogen production is regarded as a promising and efficient method due to its ability to utilize waste materials as substrates. However, despite its potential, challenges remain in optimizing the process to reduce production costs and enhance the hydrogen yield. Ongoing research focuses on developing innovative strategies, including optimizing microbial consortia and enhancing metabolic pathways, to maximize efficiency and enable large-scale implementation.^23^ Consequently, the authors aimed to evaluate the effect of ascorbic acid on hydrogen production. The study focused on determining the optimal concentration of ascorbic acid for hydrogen production by the strain Clostridium butyricum CCDBC 11 during dark fermentation, directly comparing its performance to l-cysteine—a comparison that has not been previously reported. Saccharified corn scrap (SCS), a second-generation (2G) raw material from ethanol production, was used as the substrate.

## 2 Materials and Methods

### 2.1 Inoculum and Medium

C. butyricum CCDBC 11 was utilized herein, obtained from the Milcom a.s. collection (Tábor, Czech Republic; patent no. 305450).^24^ The bacteria were cultured in RCMB medium at the following concentration per 1000 mL: 10 g of meat extract, 3 g of yeast extract, 5 g of peptone, 10 g of glucose, 1 g of soluble starch, 5 g of sodium chloride, and 3 g of sodium acetate. This formulation was previously identified as optimal for hydrogen production in an earlier study on the tested strain.^25^ The resultant RCMB medium was employed in experiments with different quantities of ascorbic acid and l-cysteine. The pH was adjusted to 7.2, and the medium was autoclaved at 115 °C for 20 min and at 225 kPa.^25^

### 2.2 Raw Material

Corn scrap is a 2G waste byproduct of the corn milling process that was discarded due to its failure to meet the quality standards required for fermentation-based bioethanol production. Due to contamination with dust and other impurities, it is no longer suitable for use in the food industry or as an animal feed. In this study, the corn meal was supplied by Ethanol Energy a.s. (Vrdy, Czech Republic) and saccharified by Novozyme enzymes sourced from the same company in accordance with the stated directions. In brief, 1 kg of corn scrap was placed in 2.45 L of water; this was then heated to 82 °C and supplemented with 0.12 g of the EN1 A Alpha Amylase enzyme. Further heating transpired, up to a temperature of 105 °C, which was maintained for 5 min; the mixture was subsequently cooled to 85 °C. Afterward, 0.12 kg of EN1 B Alpha Amylase was added to it, and stirring was performed for 2.5 h. The mixture was then topped up with water to the original volume and supplemented with the enzyme EN2 Gluco Amylase Spirizime ADV enzyme. Finally, it was stirred at 85 °C for 10 min, cooled to 30 °C, and passed through KA 0 filter paper.

#### 2.2.1 Raw Material and SCS Analysis

The moisture content of corn scrap was measured using the conventional oven-drying method, which involved drying the samples at 105 °C for 6 h.

The starch content was determined according to standard ČSN 467092-2. Corn meal was hydrolyzed by boiling with HCl (1.124% HCl) for 15 min. Clarification was performed with phosphotungstic acid (4%, 10 mL). After filtration, polarimetric measurement was conducted using an Inframatic 8600 instrument (Perten). Particle size was measured using an Analysette 3 PRO sieve shaker (Fritsch).

The physiochemical composition of the corn scrap is presented in Table 1.

**Table 1:** Physiochemical Composition of the Corn Scrap (2G Raw Material) in Dry Weight

starch (%)	moistness (%)	particle size >2 mm (%)	particle size > 1.4 mm (%)
69.40	12.49	5.83	21.26

##### 2.2.1.1 Elemental Analysis of SCS

The elemental composition of carbon, hydrogen, nitrogen, oxygen, and sulfur in the SCS samples was determined using a Flash 2000 CHNS/O+MAS200R analyzer (Thermo Scientific) via the Dumas combustion method at 960 °C. The combustion products—CO_2_, N_2_, H_2_O, and SO_2_—were transported by a helium carrier gas through a gas chromatography (GC) separation column and detected using a thermal conductivity detector (TCD). Quantification was performed using a calibration curve of standards, with identification facilitated by Eager Experience software (Thermo Scientific).

The chemical composition of SCS is presented in Table 2.

**Table 2:** Chemical Composition of SCS

	carbohydrates (g/L)		elemental analysis results (%)				
sample	glucose	fructose	C	H	N	O	S
SCS	211.6	15.5	34.8 ± 0.9	8.4 ± 0.6	3.7 ± 0.3	53.1 ± 0.9	0 ± 0

### 2.3 Experimental Procedures

The experiments were conducted at 37 °C with a glucose concentration of 10 g/L, and the pH was optimized at 7.2 for the growth of the C. butyricum CCDBC 11 strain. During hydrogen production in the fermenter, the pH was maintained at 5.6.^25^ Initially, the optimal concentration of oxygen scavengers was determined using glucose as the substrate, after which glucose was replaced by SCS. Preliminary tests were first performed with small volumes of medium in glass syringes, and the results were subsequently validated in a fermenter.

#### 2.3.1 Preliminary Tests on Optimal Concentration of Ascorbic Acid and l-Cysteine

Experiments for this purpose involved adding 10 mL of the RCMB medium into 50 mL glass syringes and applying a magnetic stirrer. The plunger of the syringes had been lubricated with paraffin oil to create a seal and reduce friction; their tips had been sealed with a rubber septum to permit sampling for gas chromatography analysis with a thermal conductivity detector (GC-TCD) during the tests. The concentrations of l-cysteine and ascorbic acid applied were 0, 0.63, 1.25, 2.5, 5, 10, 20, 40, 60, and 80 mg/L. The inoculation medium had an OD_550_ of 2.0 (a cell dry weight of 2.4 g/L), with the final concentration equaling 3%. Fermentation was performed at 37 °C and 180 rpm. Tests were performed in triplicate, each one lasting 72 h. Gas volume was measured by pushing out the plunger and reading the value on the syringe scale, with this being monitored throughout the cultivation period. Samples were collected at 30 and 72 h for GC-TCD.

#### 2.3.2 Preliminary Tests on the Optimal Concentration of SCS

Following on from those detailed above, such testing differed in that sample contained the RCMB medium in combination with SCS (instead of glucose) at concentrations (glucose present in SCS) of 0, 1.25, 2.5, 3.75, 5, 7.5, 10, and 12.5 g/L. Ascorbic acid or l-cysteine was then added to give final amounts of 0, 2.5, 5, 10, and 20 mg/L. Additionally, a mixture of both scavengers was tested at concentrations of 1.25/1.25, 2.5/2.5, and 5/5 mg/L.

#### 2.3.3 Batch Fermentation in a Fermenter for Confirmation of Results

To facilitate observation of the fermentation process in a larger medium volume (1.5 L), cultivation was conducted in a laboratory fermenter, specifically the Lambda Minifor “start-up kit” 3L (LAMBDA Instruments, Switzerland). The gas generated from the reaction in the fermenter was collected at the outlet by using the water displacement method. The gas was directed into water-filled bottles connected in parallel, with an outlet leading to a measuring cylinder monitored overnight by a camera.^12^ The pH of water was adjusted to 2 in order to minimize biogas dissolution.^26,27^ The biogas produced contains a substantial amount of carbon dioxide, which is a highly polar gas with considerable solubility in water. Upon dissolution, carbon dioxide reacts with water to form carbonic acid, which subsequently dissociates into bicarbonate (HCO_3_^–^) and carbonate (CO_3_^2–^) ions, leading to a further decrease in the water’s pH. This absorption and subsequent reaction introduce inaccuracies in the precise measurement of biogas volume.^26^ The composition of biogas was sampled periodically and analyzed by GC-TCD. The amount of CO_2_ was gauged continuously via a CO_2_ probe fitted to the fermenter. The concentrations of ascorbic acid were tested, ranging from 2.5 to 20 mg/L, and the total volume of the medium amounted to 1.5 L. The OD_550_ of the inoculation medium was 2.0 (a cell dry weight of 2.4 g/L), and the final concentration of the inoculation medium equaled 1%. The initial pH value was adjusted to 7.2, with subsequent monitoring of the fermentation medium enabled by a pH probe. During the lag phase, the pH dropped to 5.6, a level maintained by supplementation with 1 M NaOH for the remaining period of fermentation. Prior to performing the experiment, the Lambda reactor was flushed with argon gas for 15 min to remove oxygen, upon which fermentation was performed at 37 °C and 6 Hz. Liquid and gas samples were collected at frequent intervals throughout its duration.

Observation was made regarding the growth of microorganisms within the fermentation process at an optical density of OD_550_. The concentration of the biomass was determined by filtering a 5 mL sample through a 0.45 μm Millipore filter, followed by drying at 105 °C and determination of constant weight of the dried specimen.^28^ The data obtained were interpolated by applying a growth curve to a logistic model, thereby expressing the entirety of such a curve;^29^ *R* ^*2*^ values constituted a measure of the goodness of fit, as reported in charts.

### 2.4 Analytical Methods and Data Analyses

Gas chromatography was used to analyze the H_2_ and CO_2_ content. Such analyses were performed on a gas chromatograph (GC-TCD; Shimadzu GC-2010 Plus, Kyoto, Japan) equipped with a thermal conductivity detector. The subsequent data were processed using GC-Solution software. Injections were carried out manually by means of a Gastight side-hole syringe. A Carboxen 1010 PLOT column (internal diameter of 0.53 mm × 30 m long × 30 μm thick) was applied as the stationary phase. The carrier gas was argon at a flow rate of 4.99 mL/min. The volume of the sample injected was 100 μL at a split ratio of 1:5. The temperature of the injector was maintained at 200 °C. A heating cycle was conducted in the oven that commenced at 35 °C for 4.2 min, with a subsequent increase to 220 °C at 50 °C/min, which was held for 2 min; the total duration equaled 9.90 min. The temperature of the detector was set to 230 °C. Standard gases such as hydrogen, nitrogen, oxygen, carbon dioxide, and methane were purchased from Siad (Italy).

The sugar content was analyzed through high-performance liquid chromatography coupled with a refractive index detector (HPLC-RI) on a Waters Breeze QS HPLC unit (Waters, USA). Separation was achieved in a Luna NH_2_ 5 μm column (250 mm × 4.6 mm, Phenomenex USA) at 40 °C; the composition of the mobile phase comprised acetonitrile (HPLC gradient grade, VWR International s.r.o., Czechia) and water (HPLC grade, VWR) in a ratio of 80:20 (v/v). All samples and standards were passed through PES 0.45 μm syringe filters (VWR, Czech Republic) prior to injection. Each run was completed within 20 min. The data were recorded by and processed in EmPowerPro software (Waters, USA).

In order to determine the amount of volatile fatty acid (VFA) present, samples (10 mL) were centrifuged at 7500 g for 10 min, and the supernatant was subsequently filtered through a 0.22 μm syringe filter. For sample pretreatment, an inactivating acid solution was prepared using a 1:5 ratio of phosphoric acid/distilled water to minimize peak tailing caused by high temperatures.

The concentrations of VFA were determined by gas chromatography (Shimadzu GC-2010 Plus, Kyoto, Japan), on a unit equipped with a flame ionization detector and a Restek Stabilwax DA column (30 m × 0.22 mm, 0.25 μm). The temperatures for the injector and detector were set at 260 and 300 °C, respectively. Helium was applied as the carrier gas and delivered at a flow rate of 1.2 mL/min. Each sample (0.5 μL) was injected with a split ratio of 1:40. The oven initially operated at 140 °C, a temperature maintained for 14 min. Quantification was performed using calibration data obtained from standard solutions of butyric acid, acetic acid, and propionic acid diluted in distilled water at concentrations ranging from 0.1 to 10 g/L.

#### 2.4.1 Data Analysis

A modified Gompertz model (eq 1) was applied for the kinetic analysis of hydrogen production.

$$H_{H_{2}}(t)=P_{\max}\cdot \exp\left\{-\exp\left[\frac{R_{m}\cdot e}{P_{\max}}(\lambda -t)+1\right]\right\}$$ 1

where H_H_2__(*t*) is the cumulative hydrogen yield (mL), *P*_max_ represents the maximum potential of hydrogen production (mL), *R*_m_ stands for the maximum rate of hydrogen production (mL/h), *e* constitutes the Euler number, *e* = 2.71828···, λ symbolizes the lag time (h), and *t* refers to the duration of fermentation time (h).^30,31^

Statistical significance was evaluated by applying one-way ANOVA (*P* < 0.05) (OriginPro 2024 SR1, OriginLab Corporation, Massachusetts, USA); experimental data are reported as the mean values of three replicates ± standard deviation.

## 3 Results and Discussion

Preliminary experiments were conducted on a smaller scale using glass syringes for this purpose. Sealed perfectly, these were placed under optimal conditions to encourage growth and hydrogen production. Tests informed by the initial findings were then carried out in a fermenter containing 1.5 L of fermentation medium with the aim of verifying such results.

### 3.1 Effect of Oxygen Scavengers and SCS on Hydrogen Production in Preliminary Tests

#### 3.1.1 Optimal Concentration of Ascorbic Acid and l-Cysteine for Hydrogen Production

The optimal medium and glucose concentration of 10 g/L for carbon production by this strain was determined by Havlíková et al., who also discerned that the optimum pH for producing hydrogen equaled 5.6.^25^ Initial tests conducted herein verified that the same values applied under the given conditions (data not shown); hence, the optimal concentration of ascorbic acid for hydrogen production was investigated with a concentration of 10 g/L glucose and an initial pH of 7.2 to promote bacterial growth.

Although l-cysteine has been documented in the literature as an oxygen scavenger,^4,13^ its effects on the C. butyricum CCDBC 11 strain had not been previously explored. Therefore, experiments were conducted using varying concentrations of both tested oxygen scavengers.

Figure 1 shows a steep increase transpired in the hydrogen yield after adding 0.63 mg/L of such oxygen scavengers. Preliminary tests revealed a maximum of 2.24 mol H_2_/mol Glu achieved by the addition of 5 mg/L ascorbic acid after only 30 h of cultivation (Figure 1). In contrast, the control group without oxygen scavengers achieved its maximum yield only after 72 h, reaching 1.25 mol H_2_/mol Glu—44.2% lower than the maximum yield obtained with the addition of ascorbic acid. Nevertheless, the results did not indicate a statistically significant difference (*P* > 0.05) in hydrogen yield brought about by applying ascorbic acid at concentrations of 2.5–20 mg/L. The experiments with l-cysteine revealed contrasting findings since the greatest effect was observed for the amount of 5 mg/L (1.98 mol of H_2_/mol of Glu). Furthermore, supplementation with both oxygen scavengers reduced the lag phase. Samples with ascorbic acid reached a maximum yield within 30 h of incubation, while those with l-cysteine achieved 90% of the maximum yield in the same period. In contrast, samples without oxygen scavengers reached only 33% of the maximum yield during this time. These findings align with the results reported in previous studies by Bao et al., Yang and Wang, and others,^4,12,14,15^ wherein a shortening of the lag phase is reported in connection with the presence of l-cysteine. Such accelerated production and heightened yield of hydrogen, as instigated by the oxygen scavengers, could have been caused by l-cysteine and ascorbic acid reducing the value for oxidation–reduction potential (ORP) in the fermentation system, initiating cell growth as a consequence.^4^ Preliminary tests indicated that supplementing oxygen scavengers led to an earlier onset of hydrogen production. Moreover, the maximum yield with ascorbic acid supplementation was approximately 17.5% higher than with l-cysteine alone, representing a significant difference (*P* < 0.05).

**Figure 1:**
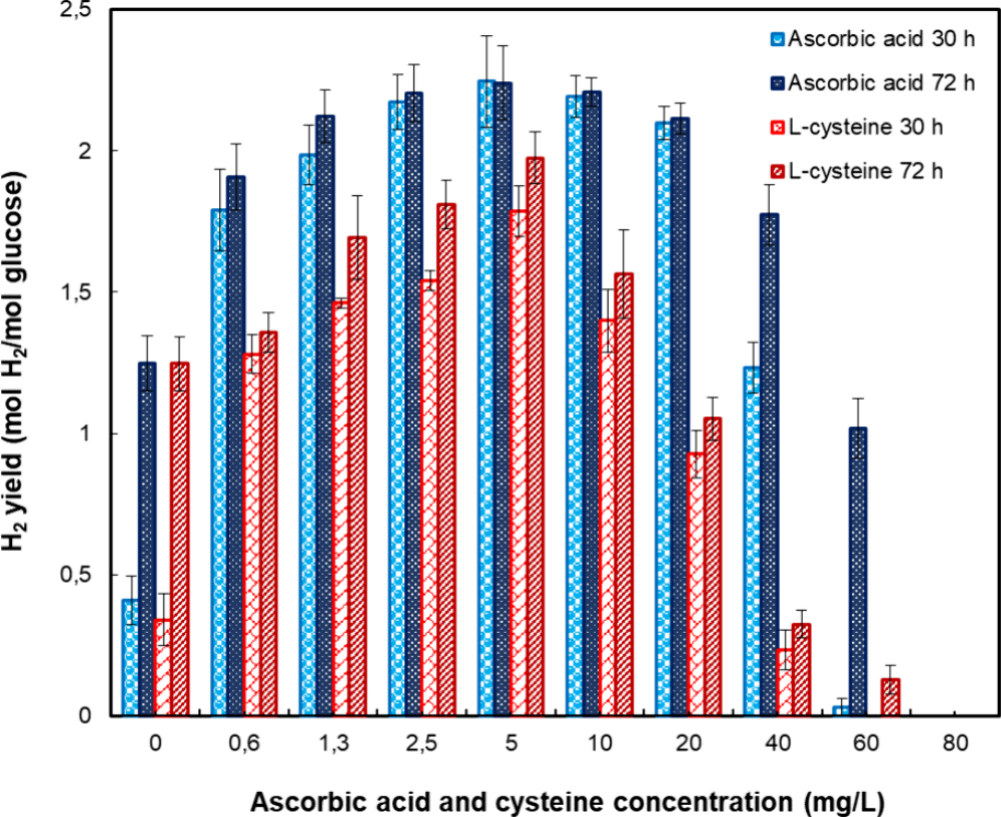
Dependence of H_2_ yield in the presence of ascorbic acid and l-cysteine across a range of concentrations: ascorbic acid (30 h cultivation), ascorbic acid (72 h cultivation), l-cysteine (30 h cultivation), and l-cysteine (72 h cultivation).

GC-TCD analysis revealed that the gas formed during fermentation contained hydrogen and carbon dioxide. The proportion of hydrogen varied between 66% and 72%, with the amount of it decreasing slightly in parallel with a rise in the biogas produced. The data indicate that neither the addition nor the type of oxygen scavenger influenced the hydrogen content in the biogas. The corresponding values are provided in the Supporting Information (Table S4).

#### 3.1.2 Optimal Concentration of SCS for Hydrogen Production

Preliminary tests were conducted to discern the optimal concentration of the substrate and oxygen scavengers in 50 mL syringes for the maximum hydrogen yield. HPLC-RI analysis showed that a concentrated solution of SCS contained 211.6 g/L glucose and 15.5 g/L fructose.

Samples were prepared for testing with glucose at concentrations of 0, 1.25, 2.5, 3.75, 5, 7.5, 10, and 12.5 g/L. The previous experiment informed which combinations and concentrations of the oxygen scavengers were selected, i.e., those demonstrating the greatest yield of hydrogen.

The highest value for hydrogen production of 2.82 mol H_2_/mol Glu was recorded after 72 h of cultivation for the sample containing 7.5 g/L glucose. This represents a significant (*P* < 0.05) increase of up to 20% compared to using glucose as the sole energy source. The maximum in this regard was observed for the sample with 5 mg/L ascorbic acid, 11% higher than that with l-cysteine (see Tables 3 and 4). Combinations of the oxygen scavengers also underwent testing, though the absolute values for hydrogen yield were below the achieved maximum. These findings indicated that mixing the two oxygen scavengers led to their effects being combined. Thus, the total optimal concentration discerned was 5 mg/L.

**Table 3:** Hydrogen Yield Engendered by Concentrations of the Substrate and Oxygen Scavengers upon 30 h of Cultivation^a^

oxygen scavengers	scavenger concentration(mg/L)	hydrogen yield(mol H_2_/mol Glu)^a^ at 30 h							
glucose concentration in SCS(g/L)		0	1.25	2.5	3.75	5	7.5	10	12.5
ascorbic acid	0	0	0	0	0	0.54	0.78	0	0
	2.5	0	1.37	1.75	1.86	2.29	2.57	1.13	0.56
	5	0	1.40	2.00	2.49	2.67	2.72	1.83	1.79
	10	0	1.33	2.03	2.13	2.27	2.30	0	0
l-cysteine	5	0	1.44	1.95	2.02	2.05	2.24	0.87	0
ascorbic acid/l-cysteine	1.25/1.25	0.52	1.52	1.98	2.01	2.15	2.26	0.14	0
	2.5/2.5	0.11	1.38	2.02	2.33	2.57	2.66	0.87	0
	5/5	0.58	1.31	1.67	1.87	2.02	2.33	1.30	0

a Relative standard deviations (RSD) for hydrogen yield ranged from 1 to 17% based on three or more measurements. The RSD values are provided in the Supporting Information File (Table S5).

**Table 4:** Hydrogen Yield Engendered by Concentrations of the Substrate and Oxygen Scavengers upon 72 h of Cultivation^a^

oxygen scavengers	scavenger concentration(mg/L)	hydrogen yield(mol H_2_/mol glu)^a^ at 72 h							
glucose concentration in SCS(g/L)		0	1.25	2.5	3.75	5	7.5	10	12.5
ascorbic acid	**0**	0	0.95	1.47	1.89	2.20	2.21	0	0
	**2.5**	0.53	1.43	1.77	1.94	2.48	2.60	2.44	0.67
	**5**	0.53	1.49	2.02	2.50	2.72	2.82	2.74	1.85
	**10**	0	1.34	2.03	2.29	2.42	2.47	2.37	0
l**-**cysteine	**5**	0.46	1.50	1.98	2.28	2.42	2.51	2.08	0
ascorbic acid/l-cysteine	1.25/1.25	0.60	1.56	2.01	2.30	2.33	2.48	2.13	0
	2.5/2.5	0.22	1.45	2.04	2.41	2.72	2.76	1.90	0
	5/5	0.60	1.35	1.85	2.08	2.15	2.36	1.70	0

a RSD for hydrogen yield ranged from 1 to 17% based on three or more measurements. The RSD values are provided in the Supporting Information Document (Table S6).

The results in Tables 3 and 4 further confirm that the addition of oxygen scavengers reduces the lag phase, particularly under optimal conditions. When 5 mg/L ascorbic acid and 7.5 g/L glucose were added, 96% of the maximum yield of 2.82 mol H_2_/mol glucose was achieved within 30 h of cultivation, whereas the control samples without oxygen scavengers reached only 35% of their final maximum yield (2.21 mol H_2_/mol Glu) in the same period.

The literature reports that raising the level of glucose increases osmotic pressure and reduces water activity, causing stress that inhibits bacterial growth and diminishes hydrogen production.^32^ These findings confirm a gradual decrease in the hydrogen yield at a higher glucose content in the medium above 7.5 g/L.

### 3.2 Effects of the Oxygen Scavengers and SCS on Hydrogen Production in the Fermenter

Throughout the tests involving the fermenter, observation was made of the cumulative volume of total biogas and the proportion of carbon dioxide. Samples were taken at selected intervals and analyzed accordingly, with GC-TCD being employed to determine the exact content of hydrogen in the gas generated, HPLC-RI evaluating the extent of carbohydrate loss, and finally, cell growth was determined.

The findings were in agreement with those of the preliminary tests, having been verified by discerning the effect of oxygen scavengers on hydrogen production in the fermenter using glucose (10 g/L) as the only energy source. The highest yield of hydrogen (1.80 mol H_2_/mol Glu) was achieved with 5 mg/L ascorbic acid. Detailed results are given in the Supporting Information File (Table S1, Figures S1 and S2).

#### 3.2.1 Effect of SCS on Hydrogen Production in the Fermenter

On the basis of preliminary research, the optimal concentrations of glucose in the saccharified corn scrap (7.5 g glu/L) and oxygen scavengers (5 mg/L) were determined and tested in a larger volume of medium (1.5 L) in the fermenter.

Peak figures for the hydrogen yield and cumulative volume were recorded for 7.5 g/L glucose in the SCS with 5 mg/L ascorbic acid (Figure 2). The cumulative hydrogen volume reached 3526 mL, corresponding to a hydrogen yield of 2.20 mol H_2_/mol Glu, 11.4% greater than in the presence of 5 mg/L l-cysteine and 40.9% higher than without the scavengers. The lag phase was significantly (*P* < 0.05) shortened by up to 65.6% with the addition of 5 mg/L ascorbic acid and by up to 38.9% with the addition of l-cysteine.

**Figure 2:**
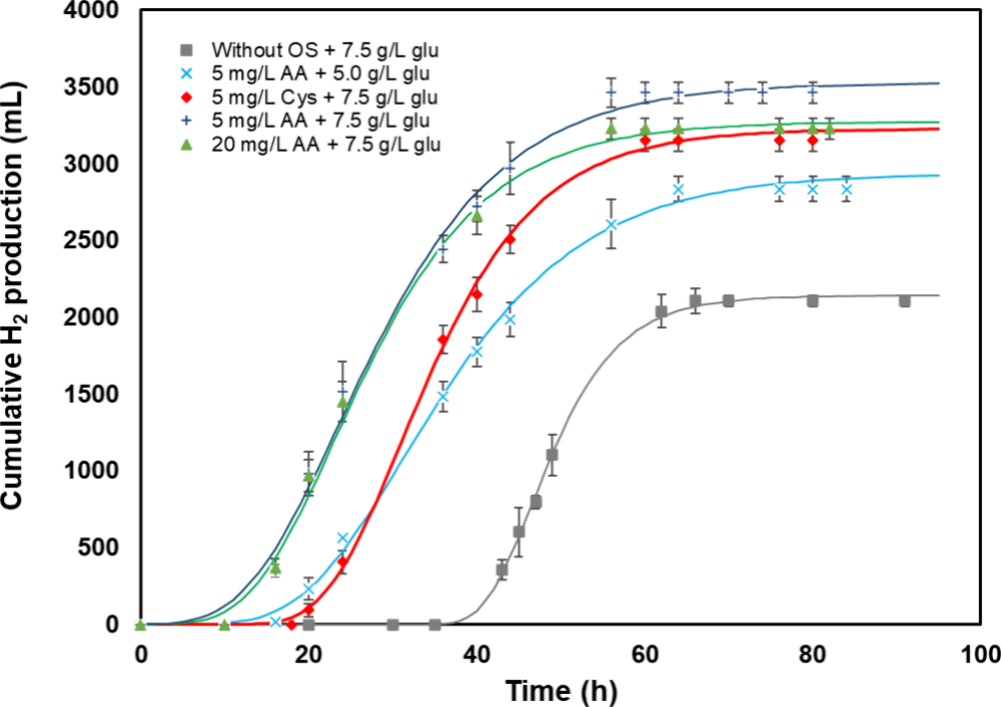
Plots for cumulative H_2_ per time for various concentrations of ascorbic acid, l-cysteine and SCS concentrations: ■, without the oxygen scavengers (7.5 g/L glucose); ◆, 5 mg/L l-cysteine (7.5 g/L glucose); × 5 mg/L ascorbic acid (5 g/L glucose); **+** 5 mg/L of ascorbic Acid (7.5 g/L glucose); and ▲ 20 mg/L ascorbic acid (7.5 g/L glucose).

The Gompertz equation coefficients for oxygen scavengers, the SCS concentration, and the hydrogen yield are summarized in Table 5.

**Table 5:** Gompertz Equation Coefficients for Various Concentrations of the Oxygen Scavengers^a^

oxygen scavengers	scavenger concentration(mg/L)	SCS(g/L)	*P*_max_ (mL)	Rm(mL/h)	λ (h)	*R* ^2^	HY (mol H_2_/mol glu)
ascorbic acid	0	7.5	2106	278	36.0	0.999	1.30
	5	5.0	2941	89	19.1	0.999	1.75
	5	7.5	3526	117	12.4	0.997	2.20
	20	7.5	3272	120	13.0	0.998	2.00
l-cysteine	5	7.5	3226	131	22.0	0.999	1.95

a HY is hydrogen yield (mol H_2_/mol glu) and λ (h) is the lag phase.

GC-TCD analysis showed that the proportion of hydrogen during fermentation ranged from 61 to 72%. Although the presence of hydrogen increased during fermentation, no difference was observed for various concentrations of the oxygen scavengers or SCS. The highest total volume of biogas (5191 mL) was achieved with the addition of 5 mg/L supplemented ascorbic acid. Hourly variations in cumulative H_2_ and CO_2_ volume for the tested concentrations of oxygen scavengers and SCS are given in the Supporting Information File (Figure S3).

Bao et al. stated the significance of l-cysteine as an important nutrient in the hydrogen production process. It acts as a bioactive agent during fermentation, facilitating interactions between bacteria and the substrate. Moreover, similar to ascorbic acid, l-cysteine acts as a reducing agent, effectively lowering the ORP in the fermentation system. This reduction in ORP enhances the growth of certain hydrogen-producing bacteria.^12^ The findings herein indicated that ascorbic acid like l-cysteine increased hydrogen production and substantially shortened the lag phase. Ascorbic acid heightened the hydrogen yield by more than 11% and reduced the lag phase by nearly 40.9% in comparison with l-cysteine.

It is not possible to contrast the peak yield recorded of 2.20 mol H_2_/mol Glu in the fermenter with the results published by Havlková et al. since they only gave proportions as percentages and did not list hydrogen yields. Several authors utilized bacteria from the genus *Clostridium* due to their high hydrogen production rates.^23,33,34^ Davila-Vazquez et al. reported a maximum hydrogen yield of 2.8 mol H_2_/mol Glu with the strain C. butyricum CM-C86.^35^ Liu et al. and Plangklang et al. achieved values of 1.15 and 1.34 mol H_2_/mol Glu, respectively, in experiments with the C. butyricum strains CGS5 and TISTR 1032.^36,37^ Herein, the C. butyricum strain demonstrated the yield of 2.20 mol H_2_/mol Glu; hence, it would appear to have potential in industrial hydrogen production.

Under optimal conditions, a highly positive effect on hydrogen production was exerted by adding ascorbic acid to the extent of 2.5–20 mg/L. Results showed that the addition of ascorbic acid reduced the lag phase and further boosted the hydrogen yield, surpassing even l-cysteine. The optimal concentration of ascorbic acid and l-cysteine for hydrogen production in a fermenter containing 1.5 L of medium was determined to be 5 mg/L. The peak yield of hydrogen determined was 2.20 mol H_2_/mol Glu, 11.4% more than with supplemented l-cysteine and 40.9% higher than without the oxygen scavengers. Moreover, the lag phase was shortened by 65.6% with the addition of ascorbic acid and by 38.9% through supplementation with l-cysteine, compared to the control sample without the oxygen scavengers. As stated above, l-cysteine is generally regarded as a low-cost additive. When applied at a concentration of 0.05 mg/L, its market price (Merck KGaA, Darmstadt, Germany) corresponds to a few U.S. dollars per 100,000 L of medium. Ascorbic acid is even more economical, costing roughly half as much. For context, the cost of hydrogen production via dark fermentation ranges from $3.20 to $48.96 per kilogram of biohydrogen.^38^

The findings revealed that using ascorbic acid instead of l-cysteine increased the yield and reduced the cultivation time for the tested strain, suggesting potential cost savings in an industrial environment. These results also suggest that the tested strain of C. butyricum CCDBC 11 shows good potential for large-scale biohydrogen production.

#### 3.2.2 Bacterial Growth in the Fermenter

The growth of microorganisms within the fermentation process was monitored using the cell weighing technique. The data obtained were interpolated through a logistic growth curve model (Figure 3). The *R* ^2^ values for all fits for the logistic model were above 0.98, i.e., an excellent match with the experimental data. Each concentration of the oxygen scavengers brought about bacterial growth, the course of which being similar in character, differing merely in the length of the lag phase. Results showed that adding between 2.5 and 20 mg/L ascorbic acid shortened the lag phase by more than half and increased the number of cells. This corresponds with findings by Bao et al., who stated that l-cysteine is an important nutrient for encouraging the growth of anaerobic bacteria.^12^ The maximum biomass concentration (12.4 g/L) was obtained with the supplementation of 5 mg/L ascorbic acid. At this concentration, the highest hydrogen yield of 2.20 mol H_2_/mol glucose was also recorded. However, further increases in ascorbic acid levels resulted in declines in both cell growth and hydrogen yield. These findings suggest that the addition of oxygen scavengers promotes cell proliferation in the C. butyricum strain CCDBC 11. The experiments demonstrated that exponential hydrogen production began when the number of cells reached the level of 4.7 to 5.0 g/L.

**Figure 3:**
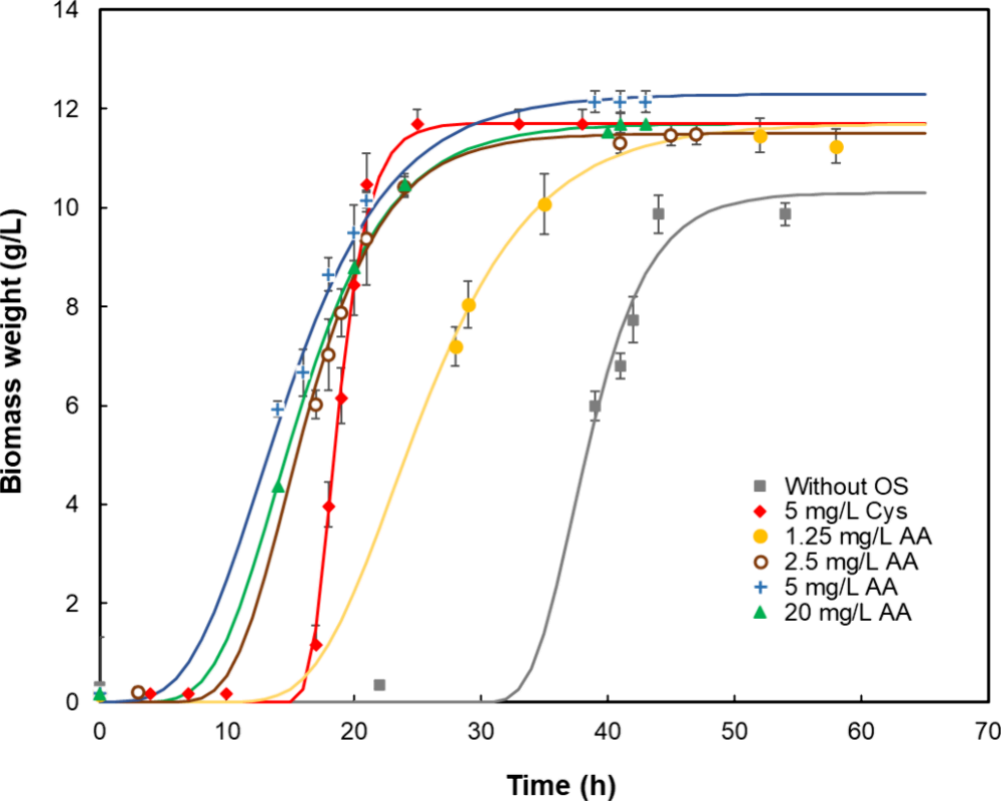
Growth curves for various concentrations of ascorbic acid and l-cysteine; ■ without the oxygen scavengers; ◆ 5 mg/L l-cysteine; ● 1.25 mg/L ascorbic acid; **○**2.5 mg/L ascorbic acid; **+** 5 mg/L ascorbic acid, ▲ 20 mg/L ascorbic acid.

Applying SCS as a substrate meant that the lag phase was shortened slightly although the number of cells did not increase (data not shown).

Although numerous studies have demonstrated the stimulatory effect of l-cysteine on hydrogen production, the underlying mechanisms of l-cysteine and ascorbic acid remain under investigation. To the best of our knowledge, only Zhao et al. examined the impact of l-cysteine supplementation on hydrogenase activity in C. beijerinckii RZF-1108. Their findings indicated that the effect of l-cysteine on hydrogen production in C. beijerinckii RZF-1108 is complex. While l-cysteine slightly enhanced *hydA* gene expression, hydrogen production was highly dependent on the interplay between cell growth and *hydA* gene expression.^15^ The influence of the environmental factor l-cysteine on enzymatic function and activity requires further investigation.

HPLC-RI analysis revealed that 0.8 g of glucose per hour was lost in the exponential phase of hydrogen production. No significant difference existed between the individual tests. When SCS was used as a substrate, only glucose was consumed, not fructose (data not shown). Although available sources suggest that C. butyricum can ferment fructose, glucose is the preferred energy source, and hydrogen production is higher when glucose is utilized. Based on these sources, we believe that the tested strain preferentially consumed glucose.^39^ However, substrate utilization was not specifically examined in this study, and whether this strain is capable of fermenting fructose remains a topic for future investigation.

#### 3.2.3 Fermentation Metabolites

Volatile fatty acids (VFAs) are important byproducts that stem from the metabolic activity of microorganisms within the production of hydrogen via fermentation. Their type and concentration directly depend on the substrate and the species of microorganism present.^30^

Table 6 shows the metabolites of C. butyricum strain CCDBC 11 formed by fermentation during hydrogen production. The results herein agree with those in the literature, indicating that the metabolic activity of hydrogen-producing acidogenic bacteria primarily gives rise to acetate and butyrate.^4,25^ The presence of butyric acid increased upon the addition of the oxygen scavengers, a finding consistent with heightened hydrogen yield and cell growth. Discerning that a greater concentration of butyric acid is brought about through supplementation with the oxygen scavengers is in agreement with other studies, wherein their positive effect on bacterial cell growth and hydrogen production is reported.^4,14^ The cause of this phenomenon is a reduction in the ORP.

**Table 6:** Final Values for Metabolites upon Applications of Oxygen Scavengers at Various Concentrations (37 °C)^a^

oxygen scavenger	concentration of oxygen scavengers(mg/L)	HY(mol H_2_/mol glu)	final ORP (mV)	acetic acid(g/L)	butyric acid(g/L)
ascorbic acid	0	0.70	–165	4.82	1.30
	1.25	1.21	–185	4.28	3.48
	2.5	1.62	–199	5.12	3.05
	5	1.80	–214	4.08	3.18
	20	1.65	–278	4.74	2.50
l-cysteine	5	1.52	–206	4.05	3.22

a HY is the hydrogen yield (mol H_2_/mol glu) and ORP is the oxidation–reduction potential.

## Supplementary Material



## Notes

Effect of varying oxygen scavenger concentrations on hydrogen production in a fermenter using glucose as substrate; highest hydrogen yield (1.80 mol H_2_/mol Glu) and shortest lag phase (13 h) obtained with 5 mg/L ascorbic acid when glucose was used (Table S1 and Figure S1); 5 g/L ascorbic acid supplementation also resulted in the shortest lag phase (13 h) and the highest biogas volume (4370 mL) (Figure S2 and Table S2); using SCS as a substrate, the addition of 5 g/L ascorbic acid leading to the highest biogas production (5191 mL) and a significant reduction in lag phase from 37 to 12 h; and hydrogen constituted 66–72% of biogas, with no significant variation across different oxygen scavenger concentrations (Table S4) (PDF)
