# Supplementary material for: Optimization of the Dark Fermentation Technique for Hydrogen Production through Supplementation with Ascorbic Acid and/or l‑Cysteine by Clostridium butyricum CCDBC 11
Source: J Agric Food Chem. 2025 May 26;73(22):13654–62. doi: 10.1021/acs.jafc.5c03194 (PMC12147110; doi:10.1021/acs.jafc.5c03194)
Supplement: Supplementary file 1 [file jf5c03194_si_001.pdf]

Supporting Information

Optimization of the Dark Fermentation Technique for Hydrogen Production through Supplementation with Ascorbic Acid and/or L-cysteine by *Clostridium butyricum* CCDBC 11

Hana Pistekova,[1*] Miroslava Dusankova,[1] Tomas Sopik,[1] Jakub Klaban,[1] Jitka Dostalkova,[1] Robert Moucka[1] and Vladimir Sedlarik[1*]

[1]Centre of Polymer Systems, University Institute, Tomas Bata University in Zlin, tr. Tomase Bati 5678,760 01 Zlin, Czech Republic

*Corresponding author: Hana Pistekova, pistekova@utb.cz; Vladimir Sedlarik, sedlarik@utb.cz

Effect of ascorbic acid on hydrogen production in the fermenter

Throughout the tests involving the fermenter, the cumulative volume of the total biogas and the proportion of hydrogen and carbon dioxide were monitored. Samples were analyzed by GC-TCD to determine the exact hydrogen content in the generated gas, HPLC-RI to evaluate the carbohydrate content, and cell growth was also monitored.

The findings were in agreement with those of the preliminary tests. The greatest cumulative volume of hydrogen (2914 mL) was achieved with supplementation of 5 mg/L of ascorbic. Glucose was applied as the substrate (Fig. 4), the aforementioned value corresponding to 1.80 mol H_2/mol Glu in hydrogen yield. This concentration also had the shortest lag phase (13 h). Adding 5 mg/L of L-cysteine brought about a drop in such volume to 2671 mL, and the associated yield decreased to 1.65 mol H_2/mol Glu. In tests without the oxygen scavengers, the latter parameter significantly ($P < 0.05$) fell by more than 60% to 0.7 mol H_2/mol Glu, confirming the results of preliminary tests in syringes. The total yield of hydrogen in the fermenter was reduced by less than 20% compared to cultivation in a syringe, hence it may be possible to discern better conditions for fermentation.

Cumulative hydrogen data were correlated with the Gompertz model (Eq. (1), and relevant coefficients (***Pmax***, ***Rm*** and ***λ***) were determined by regression analysis (Fig. 4). Table 3 summarizes the Gompertz equation coefficients for different oxygen scavengers, range of concentrations and hydrogen yield.

Figure S2 shows the hourly cumulative increase in the volume of biogas for the concentrations of the oxygen scavengers. GC-TCD analysis revealed that the biogas only contained hydrogen to the extent of 61% - 72% and carbon dioxide, in agreement with results for cumulative hydrogen. Figure S2 and Table S2 show, that the shortest lag phase (13 h) was recorded for the supplemented ascorbic acid at 5 g/L, which also exhibited the highest volume observed (4370 mL).

Effect of saccharified corn scrap (SCS) on hydrogen production in the fermenter

GC-TCD analysis revealed that the proportion of hydrogen during fermentation ranged from 61% to 72%. While the content proportion of hydrogen increased during the fermentation, no difference was observed between the different concentrations of the oxygen scavengers or SCS. The highest total volume of biogas (5191 mL) was achieved with the addition of 5 g/L of ascorbic acid, i.e. 40% higher than for the reference sample without the oxygen scavengers (Table S3, Fig. S3). The lag phase was also significantly ($P < 0.05$) shortened from 37 hours to 12 hours with supplementation of the same amount of ascorbic acid.

Table S1. Gompertz equation coefficients for the various concentrations of the oxygen scavengers

Oxygen scavengers	Scavenger concentration (mg/L)	Pmax (mL)	Rm (mL/h)	λ (h)	R^2	HY (mol H_2/mol Glu)
Ascorbic acid	0	1768	107	42.7	0.999	0.70
	1.25	2010	80	21.6	0.998	1.21
	2.50	2676	114	22.5	0.999	1.62
	5.00	2988	98	14.9	0.998	1.80
	20.00	2737	93	18.1	0.999	1.65
L-cysteine	5.00	2513	177	19.0	0.997	1.52

Where HY is hydrogen yield (mol H_2/mol Glu) and λ (h) the lag phase.

Table S2. Hourly variation in cumulative biogas for concentrations of the oxygen scavengers

Oxygen scavengers	Concentrations of oxygen scavengers (mg/L)	Total biogas (mL)	λ (h)	HY (mol H_2/mol Glu)
Ascorbic acid	0	2621±48	38	0.7
	1.25	2930±80	18	1.21
	2.5	3945±72	17	1.62
	5	4370±59	13	1.8
	20	4006±65	20	1.65
L-cysteine	5	3690±55	19	1.52

Where HY is hydrogen yield (mol H_2/mol Glu) and λ (h) the lag phase; total biogas (mL) per 1 L of medium.

Table S3. Hourly variation in cumulative biogas for the concentrations of the oxygen scavengers

SCS - glucose (g/L)	Oxygen scavengers	Concentrations of oxygen scavengers (mg/L)	Total biogas (mL)	λ (h)	HY (mol H_2/mol Glu)
7.5	Ascorbic acid	0	3160±107	38	1.30
5		5	4253±86	15	1.75
7.5		5	5159±73	11	2.20
7.5		20	4840±104	13	2.00
7.5	L-cysteine	5	4726±79	16	1.95

Where HY is hydrogen yield (mol H_2/mol glu) and λ (h) the lag phase; total biogas (mL) per 1 L of medium.

Table S4. The hydrogen content in biogas during preliminary tests using glucose as the substrate

Oxygen scavenger	Time (hours)	Oxygen scavenger concentration (mg/L)								
		0	0.6	1.3	2.5	5	10	20	40	60
Ascorbic acid	30	69.1±3.09	68.5±3.03	66.5±2.07	67.3±3.02	70.9±3.44	71.1±3.62	69.7±3.02	66.4±3.22	-
	72	67.9±2.98	66.6±2.02	65.9±3.06	66.9±2.86	70.6±3.12	70.9±3.43	67.1±2.82	66.1±3.10	67.4±1.99
Cysteine	30	70.9±2.83	67.7±3.44	66.6±3.21	66.8±3.02	65.9±3.23	68.9±3.02	67.9±3.13	67.0±3.02	-
	72	68.0±3.00	67.1±3.41	66.3±3.14	66.4±3.02	66.1±2.61	68.3±2.98	67.8±3.01	66.3±2.81	67.0±3.19

Table S5. Hydrogen yield engendered by concentrations of the substrate and oxygen scavengers upon 30 hours of cultivation

Oxygen scavangers	Scavengers concentration (mg/L)	Hydrogen yield (mol H_2/mol Glu) at 30 hours							
Glucose concentration in SCS (g/L)		0	1.25	2.5	3.75	5	7.5	10	12.5
Ascorbic acid	0	0±0.00	0±0.00	0±0.00	0±0.00	0.54±0.02	0.78±0.04	0±0.00	0±0.00
	2.5	0±0.00	1.37±0.04	1.75±0.06	1.86±0.06	2.29±0.09	2.57±0.07	1.13±0.09	0.56±0.04
	5	0±0.00	1.40±0.05	2.00±0.08	2.49±0.04	2.67±0.05	2.72±0.08	1.83±0.03	1.79±0.08
	10	0±0.00	1.33±0.03	2.03±0.06	2.13±0.09	2.27±0.06	2.30±0.16	0±0.00	0±0.00
L-cysteine	5	0±0.00	1.44±0.09	1.95±0.07	2.02±0.02	2.05±0.13	2.24±0.17	0.87±0.11	0±0.00
Ascorbic acid/ L-cysteine	1.25/1.25	0.52±0.01	1.52±0.05	1.98±0.08	2.01±0.08	2.15±0.06	2.26±0.11	0.14±0.05	0±0.08
	2.5/2.5	0.11±0.01	1.38±0.07	2.02±0.05	2.33±0.09	2.57±0.14	2.66±0.09	0.87±0.03	0±0.00
	5/5	0.58±0.03	1.31±0.05	1.67±0.09	1.87±0.08	2.02±0.06	2.33±0.08	1.30±0.10	0±0.00

Table S6. Hydrogen yield engendered by concentrations of the substrate and oxygen scavengers upon 72 hours of cultivation

Oxygen scavengers	Scavengers concentration (mg/L)	Hydrogen yield (mol H_2/mol Glu) at 72 hours							
Glucose concentration in SCS (g/L)		0	1.25	2.5	3.75	5	7.5	10	12.5
Ascorbic acid	0	0±0.00	0.95±0.04	1.47±0.02	1.89±0.06	2.20±0.03	2.21±0.11	0±0.00	0±0.00
	2.5	0.53±0.03	1.43±0.06	1.77±0.08	1.94±0.10	2.48±0.03	2.60±0.11	2.44±0.10	0.67±0.03
	5	0.53±0.03	1.49±0.07	2.02±0.10	2.50±0.06	2.72±0.04	2.82±0.12	2.74±0.16	1.85±0.08
	10	0±0.00	1.34±0.05	2.03±0.11	2.29±0.10	2.42±0.11	2.47±0.16	2.37±0.10	0±0.00
L-cysteine	5	0.46±0.04	1.50±0.06	1.98±0.10	2.28±0.06	2.42±0.05	2.51±0.11	2.08±0.11	0±0.00
Ascorbic acid/ L-cysteine	1.25/1.25	0.60±0.01	1.56±0.07	2.01±0.11	2.30±0.15	2.33±0.07	2.48±0.09	2.13±0.08	0±0.00
	2.5/2.5	0.22±0.01	1.45±0.07	2.04±0.02	2.41±0.09	2.72±0.15	2.76±0.11	1.90±0.06	0±0.00
	5/5	0.60±0.02	1.35±0.04	1.85±0.05	2.08±0.08	2.15±0.10	2.36±0.05	1.70±0.13	0±0.00

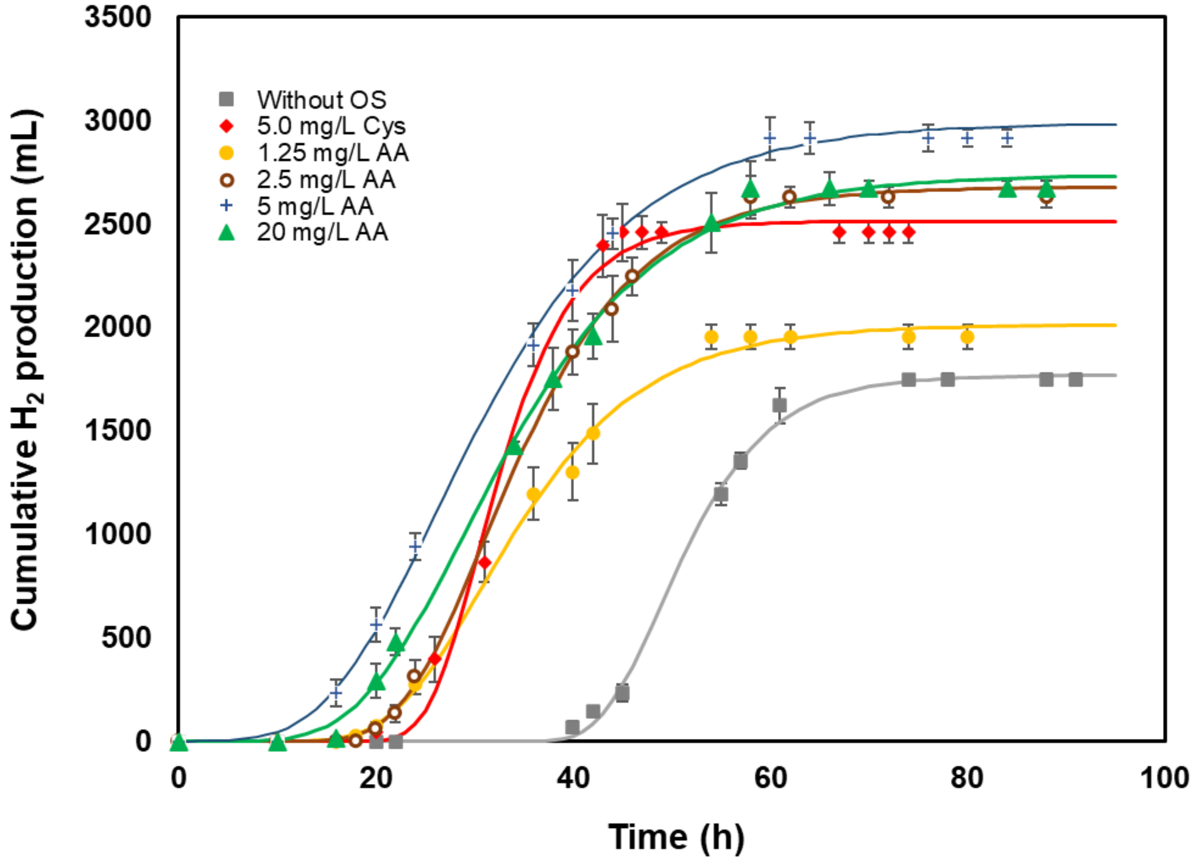

Figure S1. Plots for cumulative H_2 for the various concentrations of ascorbic acid and L-cysteine: ■ without the oxygen scavengers; ♦ 5 mg/L L-cysteine; ● 1.25 mg/L ascorbic acid; ○2.5 mg/L ascorbic acid; + 5 mg/L ascorbic acid; and ▲ 20 mg/L ascorbic acid.

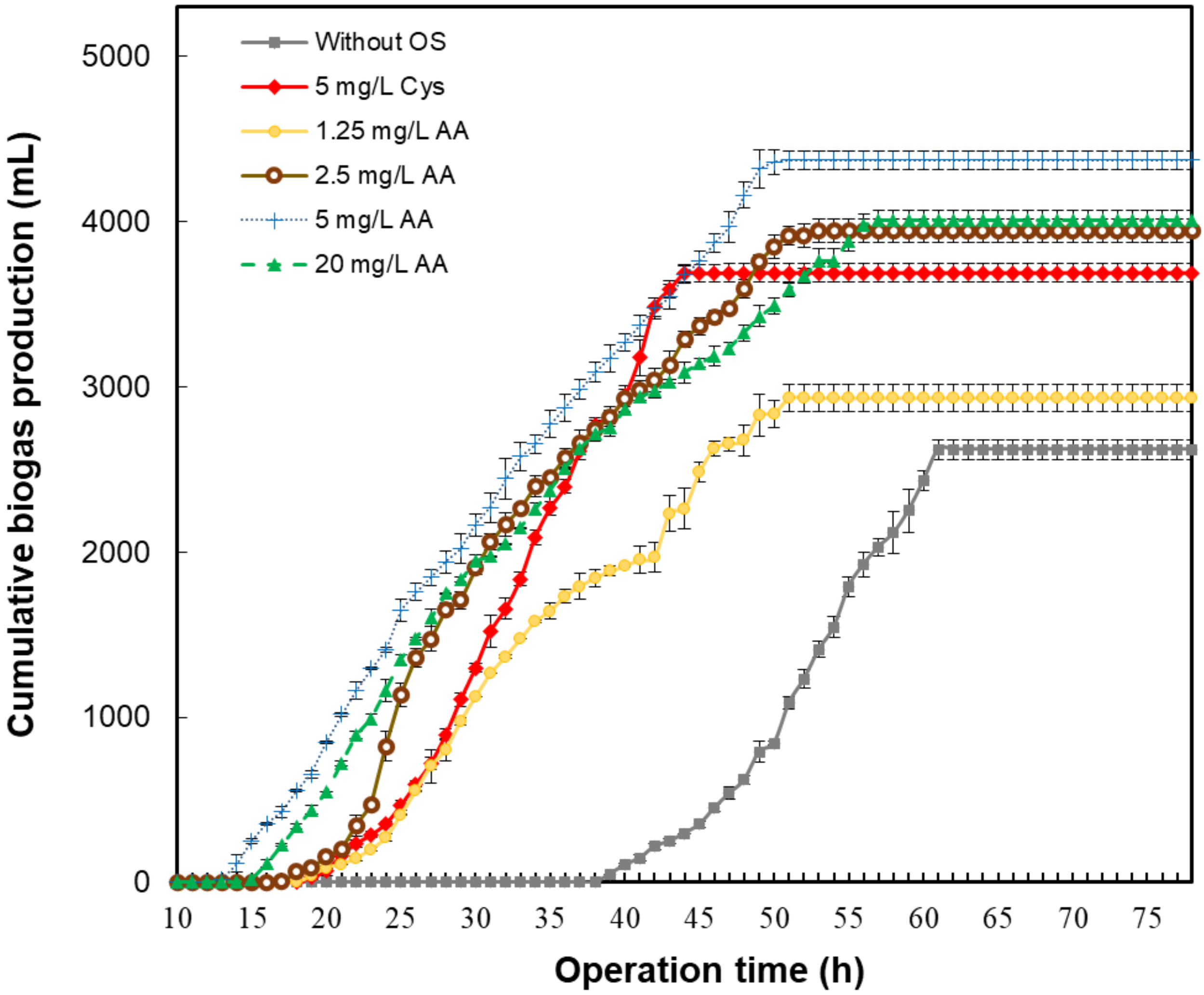

Figure S2. Hourly variation of cumulative biogas over time the concentrations of ascorbic acid and L-cysteine with glucose as energy source: ■ without the oxygen scavengers; ♦ 5 mg/L L-cysteine; ● 1.25 mg/L ascorbic acid; ○ 2.5 mg/L ascorbic acid; + 5 mg/L ascorbic acid; and ▲ 20 mg/L ascorbic acid.

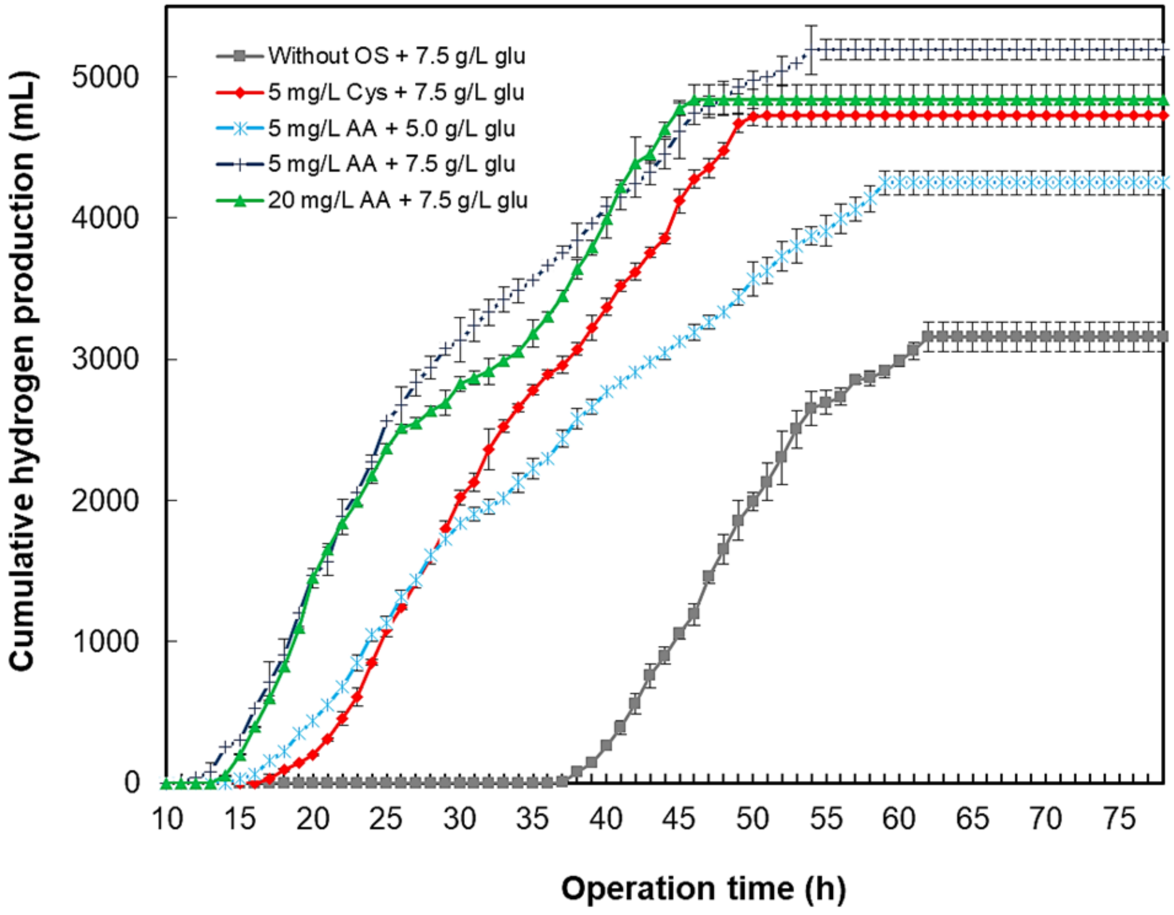

Figure S3. Hourly variation of cumulative biogas over time for the concentrations of ascorbic acid, L-cysteine and SCS: ■ without the oxygen scavengers (7.5 g/L glucose in SCS); ♦ 5 mg/L of L-cysteine (7.5 g/L glucose in SCS); x 5 mg/L ascorbic acid (5 g/L glucose in SCS); + 5 mg/L ascorbic acid (7.5 g/L glucose in SCS); and ▲ 20 mg/L ascorbic acid (7.5 g/L glucose in SCS).
